# Molecular Insights into the Role of Cysteine-Rich Peptides in Induced Resistance to *Fusarium oxysporum* Infection in Tomato Based on Transcriptome Profiling

**DOI:** 10.3390/ijms22115741

**Published:** 2021-05-27

**Authors:** Marina P. Slezina, Ekaterina A. Istomina, Tatyana V. Korostyleva, Alexey S. Kovtun, Artem S. Kasianov, Alexey A. Konopkin, Larisa A. Shcherbakova, Tatyana I. Odintsova

**Affiliations:** 1 Laboratory of Molecular-Genetic Bases of Plant Immunity, Vavilov Institute of General Genetics RAS, 119333 Moscow, Russia; omey@list.ru (M.P.S.); mer06@yandex.ru (E.A.I.); tatkor@vigg.ru (T.V.K.); konopkinalex@mail.ru (A.A.K.); 2 Laboratory of Bacterial Genetics, Vavilov Institute of General Genetics RAS, 119333 Moscow, Russia; kovtunas25@gmail.com; 3 Laboratory of Plant Genomics, Institute for Information Transmission Problems RAS, 127051 Moscow, Russia; artem.kasianov@gmail.com; 4 Laboratory of Physiological Plant Pathology, All-Russian Research Institute of Phytopathology, B. Vyazyomy, 143050 Moscow, Russia; larisavniif@yahoo.com

**Keywords:** plant immunity, cysteine-rich peptides, antimicrobial peptides, signaling peptides, elicitors, high-throughput transcriptome sequencing (RNA-seq), *Solanum lycopersicum* L., *Fusarium oxysporum* f. sp. *lycopersici*, *F. sambucinum*

## Abstract

Cysteine-rich peptides (CRPs) play an important role in plant physiology. However, their role in resistance induced by biogenic elicitors remains poorly understood. Using whole-genome transcriptome sequencing and our CRP search algorithm, we analyzed the repertoire of CRPs in tomato *Solanum lycopersicum* L. in response to *Fusarium oxysporum* infection and elicitors from *F. sambucinum*. We revealed 106 putative CRP transcripts belonging to different families of antimicrobial peptides (AMPs), signaling peptides (RALFs), and peptides with non-defense functions (Major pollen allergen of Olea europaea (Ole e 1 and 6), Maternally Expressed Gene (MEG), Epidermal Patterning Factor (EPF)), as well as pathogenesis-related proteins of families 1 and 4 (PR-1 and 4). We discovered a novel type of 10-Cys-containing hevein-like AMPs named SlHev1, which was up-regulated both by infection and elicitors. Transcript profiling showed that *F. oxysporum* infection and *F. sambucinum* elicitors changed the expression levels of different overlapping sets of CRP genes, suggesting the diversification of functions in CRP families. We showed that non-specific lipid transfer proteins (nsLTPs) and snakins mostly contribute to the response of tomato plants to the infection and the elicitors. The involvement of CRPs with non-defense function in stress reactions was also demonstrated. The results obtained shed light on the mode of action of *F. sambucinum* elicitors and the role of CRP families in the immune response in tomato.

## 1. Introduction

Crop losses due to diseases pose a serious threat to the growing human population worldwide, reducing dramatically food production [1]. Fungi destroy about 30% of crop products causing diseases [2]. Furthermore, fungi remain the main cause of biodeterioration of stored products due to the production of mycotoxins, which are fungal secondary metabolites that cannot be destroyed during food processing. Mycotoxins have been reported to be carcinogenic, hemorrhagic, and dermatitic to a wide range of organisms.

The control of fungal diseases is crucial for the maintenance of sustainable and safe food supply, and it can be achieved by improving agricultural practices, breeding for resistance, applying agrochemicals toxic to pathogens, and/or using recombinant DNA technologies [3]. Chemical control has significantly increased crop yields in recent decades; however, the emergence of resistance in pathogens and safety considerations urge the development of novel disease control strategies to overcome the pathogen threat. Biological control is regarded as an ecologically friendly strategy of disease management. Biological control agents are becoming a component of crop protection strategies that can be used in integrated plant protection programs. The modes of action of biocontrol agents include the production of antibiotics, lytic enzymes, competition for nutrients and space, and triggering induced systemic resistance in the host plant [4]. Induced systemic resistance, long-lasting resistance to a wide range of pathogens, can be initiated by the colonization of plant roots by rhizobacteria or fungi, treatment with microbial elicitor metabolites, or chemical compounds [5]. The molecular mechanisms of defense reactions evoked in plants by fungal elicitors remain largely unknown, which precludes their effective exploitation.

To protect themselves from pathogens, plants use both mechanical and chemical defenses. Plant defenses can be either constitutive or inducible. Mechanical defenses against pathogenic microorganisms and herbivores include thick waxy cuticles, cell walls, and special adaptations such as trichomes, thorns, and spines, which prevent the penetration of plant tissues. Chemical defenses that come into play if mechanical barriers had been breached involve compounds toxic or repellent to the invaders [6]. Among them are secondary metabolites (phytoanticipins and phytoalexins) and polypeptide-based molecules—defense proteins and peptides. Most plant defense peptides belong to cysteine-rich peptides (CRPs) containing from 4 to 12 cysteine residues that are engaged in disulfide bonding. They are classified into families according to the number of cysteine residues, their arrangement in the polypeptide chain (the so-called cysteine motif), amino acid sequence, and 3D structure similarity. Defense peptides encompass antimicrobial peptides (AMPs), enzyme inhibitors, and signaling peptides. Some reported CRPs are involved in developmental processes, and their role in immune processes remains elusive [7]. Dozens of CRP genes have been predicted in plant genomes by in silico mining [8]. However, their role in induced resistance mechanisms remains poorly understood.

Tomato belongs to widely cultivated vegetables worldwide and represents a vital constituent of human diet enriched in antioxidant compounds, which decrease the risk of cardiovascular diseases and cancer [9]. It is a diploid species (2*n* = 12) with a relatively small genome (around 950 Mb). Tomato wilt caused by *Fusarium oxysporum* f. sp. *lycopersici* is one of the main diseases of tomato plants, reducing both the quantity and quality of the production. The fungus penetrates the epidermis of roots, spreads through the vascular tissue, and colonizes the xylem vessels, causing water stress followed by wilting of the host plants [10]. In addition, the pathogen produces toxins, such as fusaric acid, fusarins, dehydrofusaric acid, moniliformin, and others that play a role in the development of wilt symptoms and disease progression [11].

Earlier, we reported that *Fusarium sambucinum* strain FS-94 protected tomato plants against vascular wilt of tomato. Seed soaking or immersion of seedling roots in the FS-94 spore suspension prior to inoculation with the pathogenic *F. oxysporum* f. sp. *lycopersici* isolates significantly decreased wilt symptoms and delayed their development [12]. Only viable germinating FS-94 conidia, in contrast to the spores killed by autoclaving, displayed the protective activity, suggesting that FS-94 produces metabolites that elicit disease resistance. We further showed that induced resistance (IR) developed due to activation and priming of the salicylic acid-dependent signaling system [12]. However, the role of polypeptide-based defense compounds in IR development has not been elucidated.

The goal of this work was to explore the role of different families of CRPs in immune response to *F. oxysporum* infection and resistance mechanisms induced in tomato plants by the elicitor metabolites of *F. sambucinum* FS-94 using high-throughput transcriptome sequencing (RNA-seq). By global transcriptome profiling, we identified genes encoding CRPs in tomato plants and monitored changes in their expression levels induced by the pathogenic *F. oxysporum* strain F37, FS-94 elicitors, and by the pathogen in elicitor-pretreated plants. To the best of our knowledge, this is the first report on transcriptional profiling of different CRP families is response to fungal infection and biogenic elicitors. As a result, we revealed specific sets of up- and down-regulated peptide genes associated with pathogenesis and induced resistance development. The results obtained shed light on the molecular basis of the immune response activated by *F. oxysporum* infection and elucidate the mode of action of *F. sambucinum* elicitors, paving the way for their exploitation as a biocontrol agent in agriculture to fight Fusarium wilt. Furthermore, in transcriptomic data, we discovered a novel gene, encoding a precursor of a previously unknown type of a 10-Cys-containing hevein-like peptide and revealed its close homologues in Solanaceae species by in silico mining, thus expanding the repertoire of CRP genes present in plant genomes.

## 2. Results

### 2.1. F. sambucinum FS-94 Elicitors Protect Tomato Seeds and Seedlings from F. oxysporum F37 Infection

We carried out plant protection assays with tomato seeds and seedlings using different concentrations of the elicitor fraction at 5 and 10 days post inoculation (dpi) with the pathogenic *F. oxysporum* strain F37. The results are shown in Table 1. They clearly demonstrate that treatment of tomato seedling roots with the protein-containing fractions isolated from the purified extract of FS-94 mycelium, which was shown to be non-toxic for *F. oxysporum* [13], resulted in significant reduction in disease severity on young plants (Table 1). Non-protein fractions were inactive; the protective effect of the elicitors disappeared if the extract was incubated at 100°C for 10 min prior to treatment [13].

### 2.2. Transcriptome Sequencing and Assembly

To study transcriptional changes in CRP genes, 10 cDNA libraries were sequenced on HiSeq4000 instrument (Illumina, San Diego, CA, USA). These cDNA libraries included two biological replicates obtained from control and challenged with *F. oxysporum* at two time points (2 and 4 dpi) plants, elicitor treated, and elicitor pretreated and infected plants (Table 2).

Raw reads obtained from the sequencer were preprocessed using Trimmomatic software. During the preprocessing stage, the removal of technical sequences and quality trimming were performed. The number of clean reads remaining for each library after preprocessing is shown in Supplementary Materials Table S1. Combined reads from all libraries were assembled using transcriptome assembler software rnaSPAdes [14]. As a result, 137158 contigs were assembled. A search for CDS in the assembled contigs resulted in 45095 coding sequences. Length statistics of raw assembled contigs and CDS are shown in Supplementary Materials Table S2.

### 2.3. Identification of CRP Precursors in Tomato Transcriptomes

Using our earlier developed pipeline for CRP mining in transcriptome datasets, we discovered as many as 106 transcripts encoding precursors of cysteine-rich peptides and short cysteine-rich proteins. They belonged to different known families of defense peptides: defensins, non-specific lipid-transfer proteins, snakins, thionins, hevein- and knottin-like peptides, signaling peptides (RALFs) and peptides whose functions are not clearly understood (MEG and Ole e1 and 6), and PR-1 and 4 family proteins. One CRP with a previously unknown cysteine motif was also found.

#### 2.3.1. Defensins

Defensins were discovered in all multicellular organisms [15]. Amino acid sequences of plant defensins are highly variable, while the fold of the molecule stabilized by four or five (in floral defensins) disulfide bonds is similar and comprises the so-called cysteine-stabilized αβ-motif (СSαβ-motif) with an α-helix running parallel to the triple-stranded β-sheet [15]. According to Silverstein et al. [9], the cysteine motif of classical defensins is as follows: CX{4,25}CX{2,12}CX{3,4}CX{3,17}CX{4,32}CXCX{1,6}C. The biological activities of plant defensins are mainly associated with defense functions. They inhibit the growth of fungi, bacteria, or herbivores, disturbing the membranes of the pathogens or acting as enzyme inhibitors or ion channel blockers. The role of defensins in abiotic stress tolerance and development was also demonstrated [15]. Plant defensins are synthesized as precursor proteins containing a signal peptide transferring the mature peptide to the apoplast. Precursors of floral defensins possess a C-terminal prodomain that targets them to the vacuole and masks the toxicity of the mature peptide.

**Figure 1:**
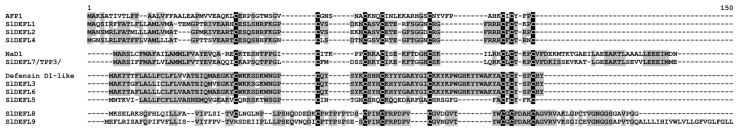
Multiple sequence alignment of *S. lycopersicum* DEFL precursors and *Arabidopsis thaliana* AFP1 (NP_565119.1), *Nicotiana alata* NaD1 (AAN70999.1), and *Solanum pennellii* defensin D1-like (XP_015081705.1). Cysteine residues are shaded black, and identical amino acids are shaded gray.

In tomato transcriptomes, nine sequences encoding defensin-like (DEFL) precursors with an 8-cysteine motif of classical defensins were discovered (Figure 1, Supplementary Materials Table S3). They consist either of a signal peptide and a mature peptide region (SlDEFL1−6) or contain an additional C-terminal prodomain (SlDEFL7−9). Seven polypeptides are basic and two are acidic (Supplementary Materials Table S3). Of DEFL precursors, seven were annotated as defensin-like sequences and two (SlDEFL8 and 9), as uncharacterized proteins. In SlDEFL9, the first cysteine in the motif has mutated to arginine. In *S. lycopersicum* genome, we have predicted 34 DEFL sequences possessing the 8-cysteine motif typical for classical defensins [16]. Thus, only a portion of defensin-like genes is expressed under our experimental conditions. The discovered sequences form groups of related peptides with high sequence similarity (Figure 1, Supplementary Materials Table S3).

It is of particular interest that three tomato defensins (SlDEFL1, 2 and 4), which form a separate clade on the phylogenetic tree (Supplementary Materials Table S3), possess an identical pentapeptide RGFRRR in the γ-core region associated with antimicrobial activity of plant defensins [17]. This short peptide alone was shown to exhibit antifungal activity [18]; therefore, tomato defensins harboring this motif are antifungal.

**Figure 2:**
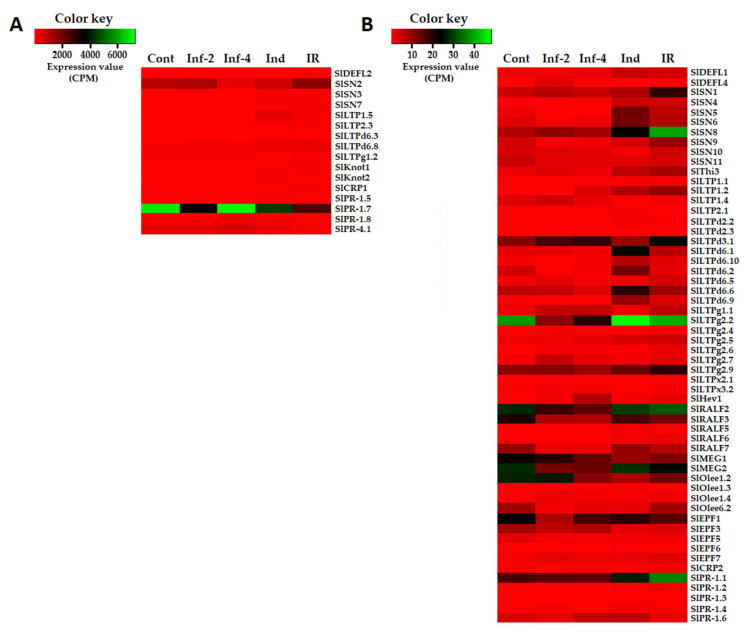
Heatmaps of differentially expressed CRP genes. (**A**) Genes with expression levels above 50 CPM (Counts per Million Mapped Reads) at least in one transcriptome. (**B**) Genes with expression levels below 50 CPM in all transcriptomes. Cont, Inf-2, Inf-4, Ind, and IR designate control, infected at 2 or 4 dpi, induced and IR-displaying plants, respectively.

Only these three genes encoding defensin-like precursors SlDEFL1, 2, and 4 were differentially expressed in tomato plants infected with *F. oxysporum* and/or treated with *F. sambucinum* elicitors (Supplementary Materials Table S4). Of them, the expression level of SlDEFL2 was the highest (Figure 2). The SlDEFL1 gene was weakly expressed in control plants, but it was up-regulated in elicitor-treated and IR-displaying plants (Figure 2, Supplementary Materials Table S4). The SlDEFL2 gene behaved similarly: it was insensitive to infection but was up-regulated by the elicitors and in IR-displaying plants. The SlDEFL4 gene was poorly expressed in healthy plants but was highly up-regulated by infection both at 2 and 4 dpi.

#### 2.3.2. Non-Specific Lipid-Transfer Proteins

Non-specific lipid-transfer proteins (nsLTPs) are found in all land plants [19]. They are characterized by a conserved 8-cysteine motif: CX{6,15}CX{9,31}CCX{8,21}CX CX{13,35}CX{5,18}C. Originally, nsLTPs were divided into two groups according to molecular weight [20]. Class 1 LTPs comprised polypeptides, whose molecular weight was ≈9 kDa, and class 2 included polypeptides with molecular weight of ≈7 kDa. Later, the nsLTP classifications took into account the spacings between the cysteine residues and the number and position of introns in the nsLTP genes [19]. The fold of the nsLTP molecule includes four or five parallel α-helices stabilized by four disulfide bridges forming a tunnel-like cavity where the binding of lipids occurs. Similarly to defensins, nsLTPs are produced as precursor proteins containing a signal peptide and a mature peptide domain. In some nsLTPs, a GPI (glycosylphosphatidylinositol) anchor is present. nsLTPs are involved in a variety of functions: defense against biotic and abiotic stresses, synthesis of polymers, such as suberin and waxes, pollen and seed development, cell elongation, and nodule formation.

**Figure 3:**
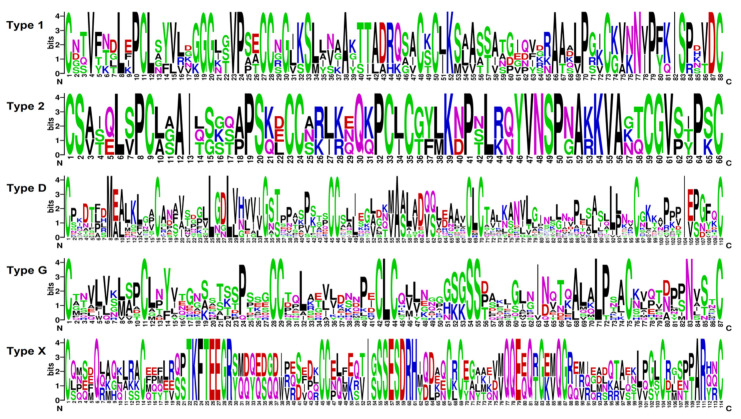
Sequence logo plots of aligned 8-cysteine motif sequences for each SlLTP type. The numbers on the x-axis represent the positions in the 8-cysteine motif. On the y-axis, the information content measured in bits is presented.

In tomato transcriptomes, we discovered 44 sequences with the 8-cysteine motif characteristic of plant nsLTPs and annotated as predicted non-specific LTPs and 14-kDa proline-rich proteins (Supplementary Materials Table S3). The three polypeptides SlLTPd3.2, SlLTPx1.1, and SlLTPx2.1 were annotated as uncharacterized proteins of *S. lycopersicum*. All discovered sequences possessed the domain specific for lipid-transfer proteins, as revealed by InterProScan [21]. Note that 81 sequences of nsLTP-like precursors were found in tomato genome by database search [16]. nsLTPs discovered in tomato transcriptomes include basic, neutral, and acidic polypeptides. Of them, seven sequences were new, showing less than 100% sequence identity to nsLTPs from *S. lycopersicum* present in NCBI databases. According to the earlier developed classification [22], which takes into account spacings between the adjacent cysteine residues, the detected sequences were grouped into Types 1, 2, D, G, and X (Figure 3, Supplementary Materials Table S3). All putative nsLTPs are produced as precursor proteins containing a signal peptide and a mature domain. Some sequences possess additionally a GPI anchor. On the phylogenetic tree based on nsLTP precursor sequences, several clusters are seen: Type 1 and 2 sequences form separate clusters, Type G separates into two clusters, Type D polypeptides form one main cluster and several small subclusters, and Type X sequences comprise four sequences showing low similarity with each other (Supplementary Materials Table S3).

**Figure 4:**
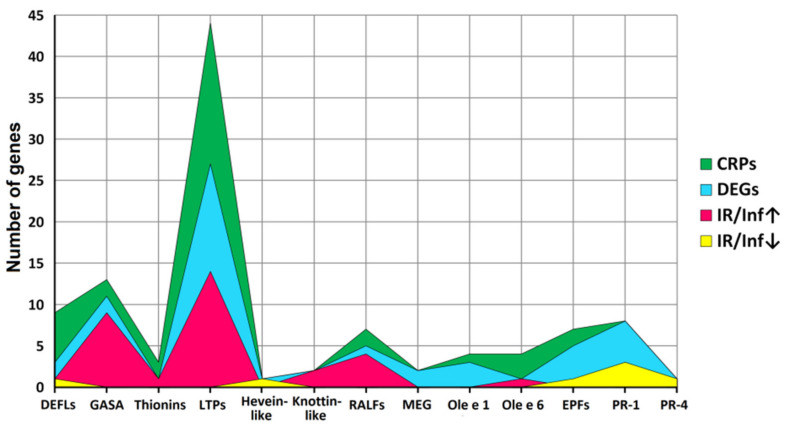
Family distribution of CRP genes, differentially expressed genes (DEGs), and genes up-regulated (IR/Inf↑) and down-regulated (IR/Inf↓) in IR-displaying plants compared to infected at 4 dpi.

Twenty-seven nsLTP genes showed differential expression under the experimental conditions used (Figure 4, Supplementary Materials Table S4). Several nsLTP genes were highly expressed in tomato transcriptomes: SlLTP1.5, SlLTP2.3, SlLTPd6.3, SlLTPd6.8, and SlLTPg1.2 (Figure 2A). The expression level of other nsLTP genes was relatively low (Figure 2B). Different treatments induced specific transcriptional changes (Figure 2). Thus, infection with *F. oxysporum* up-regulated five genes at 2 dpi and six genes at 4 dpi, and it down-regulated six genes at 2 dpi and five genes at 4 dpi (Supplementary Materials Table S5). Treatment with the resistance inducers up-regulated many more genes (15) and down-regulated only two genes. Challenge inoculation with the pathogen of elicitor-pretreated plants also considerably activated the expression of nsLTP genes: 18 nsLTP genes were up-regulated, and only one gene was down-regulated (Supplementary Materials Table S5). Two genes were up-regulated both by infection and elicitors (SlLTP1.2 and SlLTP2.3), some genes were specifically induced by infection (e.g., SlLTPg1.1 and SlLTPx2.1), while others were specifically induced by the elicitors (e.g., SlLTPd6.1, SlLTPd6.9). In IR-displaying plants, a lot of LTP genes were up-regulated in comparison with infected plants without elicitor pretreatment (Figure 4, Supplementary Materials Table S6).

#### 2.3.3. Snakin/Gibberellic Acid Stimulated-like (GASA)

Snakins belong to cysteine-rich AMPs, whose molecules consist of approximately 65 amino acid residues containing as many as 12 cysteine residues and forming the motif as follows: CX{3}CX{3}CX{7,11}CX{3}CX{2}CCX{2}CX{1,3}CX{11}CX{1,2}CX{11,14}-KCP. These AMPs are synthesized as precursors, pre- or preproproteins. Snakin genes are responsive to stressful factors and are up-regulated by gibberellins [23]. They suppress the growth of pathogenic bacteria and fungi in vitro [24]. The overexpression of snakin genes in transgenic plants results in increased pathogen resistance [25]. Accumulating evidence indicate that besides defense, snakins are involved in developmental processes [26].

**Figure 5:**
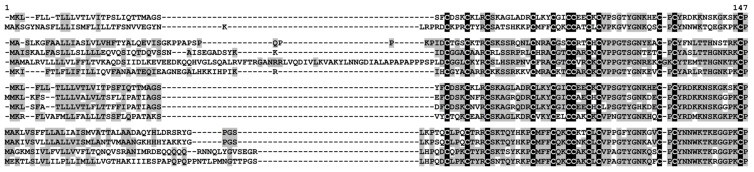
Multiple sequence alignment of *S. lycopersicum* snakin precursors and *Solanum tuberosum* SN1 (Q948Z4.1). Cysteine residues are shaded black, and identical amino acids are shaded gray.

In tomato transcriptomes, we found 13 sequences of snakin precursors (Figure 4 and Figure 5, Supplementary Materials Table S3). All of them are annotated as gibberellin-regulated proteins, and all but one polypeptide showed 100% identity to the gibberellin-regulated proteins from *S. licopersicum*. In the genome, 17 sequences of snakin-like peptides were discovered [16], so the vast majority of snakins were expressed under our experimental conditions. All discovered snakins are basic. In total, eleven snakin genes were found to be responsive to infection, and/or elicitor treatment (Figure 2, Supplementary Materials Table S4). Of them, three genes were highly expressed in tomato transcriptomes (SlSN2, SlSN7, SlSN3), two genes (SlSN1, SlSN8) were moderately expressed, while the expression levels of the remaining genes were lower (Figure 2). The SlSN2 gene was among the most abundantly expressed in all four transcriptomes, especially in healthy, infected (2 dpi) tomato plants and in the plants displaying IR. The elicitors and *F. oxysporum* infection of elicitor-pretreated plants enhanced expression of the highly expressed snakin genes SlSN7 and SlSN3.

Infection down-regulated the expression of snakin genes: six genes were down-regulated at 2 dpi and five genes were down-regulated at 4 dpi (Supplementary Materials Table S4 and S5). The elicitors up-regulated six snakin genes and down-regulated only two. Infection of elicitor-pretreated plants up-regulated eight genes, while no snakin genes were down-regulated (Figure 4). Hence, positive correlation between the expression of snakin genes and elicitation of defense response and resistance becomes obvious (Figure 2, Supplementary Materials Table S5).

#### 2.3.4. Thionins

Thionins are AMPs with six or eight cysteine residues engaged in disulfide bonds, which were found in many plant species. Their 3D structure resembles the letter L, in which the long arm is formed by two antiparallel α-helices, and the short arm is represented by two parallel β-strands [27]. Thionins are toxic to fungi, bacteria, and insects. Their toxicity is due to the formation of pores in membranes. Similar to other AMPs, thionins are synthesized as precursors containing a signal peptide, a mature peptide domain, and a C-terminal prodomain with six conserved cysteine residues necessary for vacuole transport.

**Figure 6:**
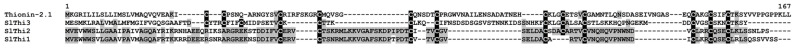
Multiple sequence alignment of *S. lycopersicum* thionin precursors and *Arabidopsis thaliana* thionin-2.1 (NP_565038.1). Cysteine residues are shaded black, and identical amino acids are shaded gray.

Three thionin-like sequences were discovered in tomato transcriptomes, two of which (SLThi2 and SlThi3) showed 100% sequence similarity to uncharacterized proteins of *A. thaliana* and a thionin-like protein of *S. lycopersicum*, respectively, while one sequence SlThi1, which showed 97% similarity to the uncharacterized protein of *A. thaliana* XP_004237990.1, lacked a cysteine residue in the conserved 6-Cys motif of the C-terminal domain of the precursor due to deletion (Figure 6, Supplementary Materials Table S3). All discovered sequences were assigned to thionin-like proteins by InterProScan [21]. The mature peptides are basic and predicted to be AMPs.

Of the discovered thionin-like genes, SlThi1 and SlThi2 genes were irresponsive to the experimental treatments. Conversely, Thi3 was up-regulated both by the elicitors and infection of elicitor-pretreated plants (Figure 2, Supplementary Materials Table S5).

#### 2.3.5. Hevein-Like Peptides

Hevein-like peptides were named after hevein, which is an antimicrobial peptide from the rubber tree latex. These small (approximately 40 amino acid residues), cysteine-rich peptides differ in the number of cysteine residues in the molecule (6, 8, or 10) [28]. Only three types of 10-Cys-containing hevein-like AMPs have been reported so far: EAFP1 and EAFP2 from the bark of *Eucommia ulmoides* Oliv. [29], Ee-CBP from the bark of the spindle tree *Euonymus europaeus* L. [30], and WAMPs from wheat *Triticum kiharae* Dorof. & Migush. [31]. The spatial structure of hevein-like AMPs is well conserved and includes an α-helical region and three or four antiparallel β-strands. All hevein-like peptides contain a chitin-binding site consisting of several conserved hydrophobic amino acid residues and a serine residue that bind with the chitin of the cell walls of pathogenic fungi. It is commonly accepted that the binding of hevein-like peptides to the tips of growing fungal hyphae interferes with their elongation, resulting in the suppression of fungal growth [32]. However, for the wheat 10 Cys-containing hevein-like peptides WAMPs, it was shown that they are specific inhibitors of fungalysin, which is a secreted metalloproteinase of *Fusarium* fungi that targets plant defense chitinases [33]. Hevein-like AMPs are synthesized as preproproteins. Some peptides of this family are produced during the proteolytic processing of class 1 chitinases [32].

**Figure 7:**
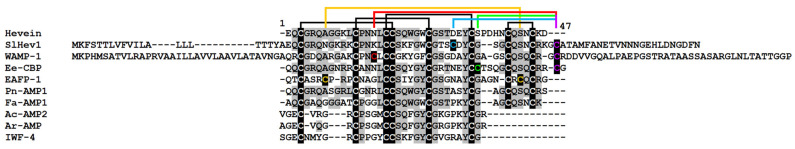
Multiple sequence alignment of hevein-like peptide precursors of *S. lycopersicum* SlHev1 and *T. kiharae* WAMP-1 (P85966.2), hevein of *Hevea brasiliensis* (1Q9B_A) and hevein-like peptides from *Pharbitis nil* Pn-AMP1 (P81591), *E. ulmoides* EAFP1 (P83596), *E. europaeus* Ee-CBP (Q7Y238), *Fagopyrum esculentum* Fa-AMP1 (P0DKH7), *Amaranthus caudatus* Ac-AMP2 (Q9S8Z7), *A. retroflexus* Ar-AMP (Q5I2B2), and *Beta vulgaris* IWF4 [34]. Cysteine residues are shaded black, and identical amino acids are shaded gray. The arrangement of disulfide bonds is shown above the alignment.

**Figure 8:**
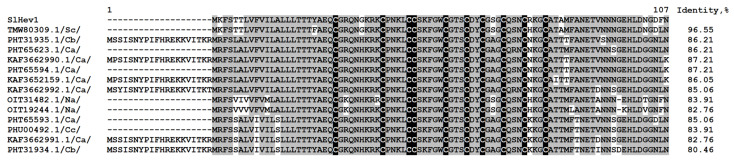
Multiple sequence alignment of *S. lycopersicum* hevein-like peptide precursor SlHev1 and homologous sequences of *Solanum chilense* (Sc), *Capsicum baccatum* (Cb), *C. annuum* (Ca), *Nicotiana attenuata* (Na), and *C. chinense* (Cc). Cysteine residues are shaded black, and identical amino acids are shaded gray. Sequence identity values are shown to the right of the alignment.

One polypeptide encoding a precursor of a hevein-like peptide named SlHev1 was discovered in tomato transcriptomes (Supplementary Materials Table S3). It contains 10 cysteine residues arranged in a novel previously undescribed motif. Thus, it is the fourth type of 10-Cys motifs reported for plant hevein-like AMPs. Amino acid alignment of SlHev1 with other 10 Cys-containing hevein-like AMPs is presented in Figure 7. Interestingly, homologous peptides with extremely high sequence similarity to SlHev1 were discovered by in silico mining in other Solanaceae species pointing to the vital role of this peptide in plant’s physiology (Figure 8). The peptide is poorly expressed in control plants but is strongly up-regulated by infection, elicitors, and in IR-displaying plants (Figure 2, Supplementary Materials Table S4).

**Figure 9:**
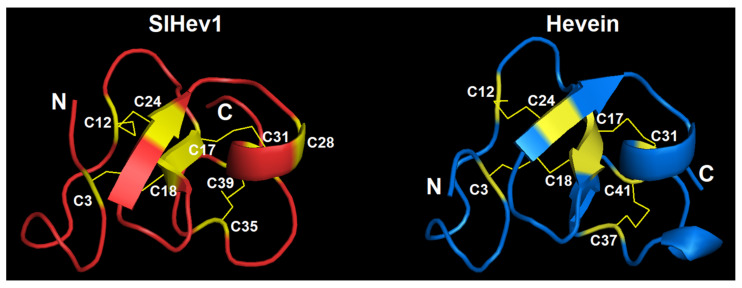
The 3D structure model of SlHev1 and the NMR solution structure of hevein (1HEV). Modeling was carried out using SWISS-MODEL [35]. Disulfide bonds are shown by thin yellow lines. The N-and C-termini are indicated by N and C, respectively. Cysteine residues are colored yellow and numerated according to their position in the polypeptide chain.

We modeled the 3D structure of the SlHev1 mature peptide with the SWISS-MODEL program using the crystal structure of the chitin-binding module (CBM18) of a chitinase-like protein from *Hevea brasiliensis* (4MPI) as a template (Figure 9). The template was chosen with the highest GMQE score (Global Model Quality Estimation) of 0.72, which combines properties from the target-template alignment and the template structure. The global fold of the SlHev1 mature peptide is quite similar to that of other hevein-like peptides and hevein itself. The structural model contains two antiparallel β-strands and one α-helix connected by four disulfide bridges (C3-C18, C12-C24, C17-C31, and C35-C39). The fifth disulfide bond is absent due to incomplete coverage (only first 42 amino acid residues without the last cysteine residue) and the absence of a structure template with a similar cysteine motif.

#### 2.3.6. Knottin-Like Peptides

In the molecules of knottin-like peptides, six cysteine residues form the cysteine motif as follows: СX{3}СX{5}СX{5}СX{2}СX{6,13}С. All cysteine residues are engaged in three disulfide bonds, and one of them “penetrates” the ring formed by the other two disulfide bonds [36]. This structure is called the cystine knot. It gives the knottin molecules extraordinary stability to proteolytic degradation and extreme temperatures. The three-dimensional structure of knottins involves three antiparallel β-strands and their connecting loops [37]. Knottins are synthesized as preproproteins. They display various biological activities including antifungal, insecticidal, and anticarcinogenic [36]. The knottins of Solanaceae plants act as carboxypeptidase inhibitors [38].

**Figure 10:**
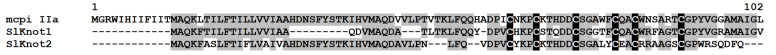
Multiple sequence alignment of *S. lycopersicum* knottin precursors and *S. tuberosum* metallocarboxypeptidase inhibitor precursor (mcpi) IIa (NP_001275048.1). Cysteine residues are shaded black, and identical amino acids are shaded gray.

Two knottin precursors SlKnot1 and SlKnot2 were discovered in tomato transcriptomes (Supplementary Materials Table S3). They showed 100% sequence identity to the metallocarboxypeptidase inhibitors from *S. lycopersicum*. They also showed sequence similarity to a metallocarboxypeptidase inhibitor from *S. tuberosum* (Figure 10). The expression levels of SlKnot1 and SlKnot2 varied considerably in tomato plants depending on the treatment (Figure 2). Infection with *F. oxysporum* suppressed expression of the SlKnot1 gene, while it up-regulated the SlKnot2 gene (Table S4, Supplementary Materials Table S5). However, infection of elicitor-pretreated plants up-regulated both SlKnot genes, though, much more significantly, it enhanced expression of the SlKnot2 gene (Supplementary Materials Table S4). The SlKnot2 gene was also significantly up-regulated in elicitor-treated plants.

#### 2.3.7. RALFs

RALFs are 5-kDA peptides that cause rapid alkalinization of the medium upon addition to cell culture. They were first discovered in tobacco [39] and later in other plant species. These peptides are derived from the C-terminal region of the precursor protein. The mature peptide possesses four cysteine residues arranged in a motif CX{3,12}CX{5,21}CX{5,6}C, which form two disulfide bridges. Cysteines were postulated to be vital for the biological activity of RALFs [39]. RALFs possess several other conserved oligopeptide motifs necessary for receptor binding [40]. RALFs belong to signaling peptides involved in plant growth and development. They were shown to inhibit root hair growth and nodule development, control the growth of pollen tubes, and regulate plant immune signaling.

**Figure 11:**
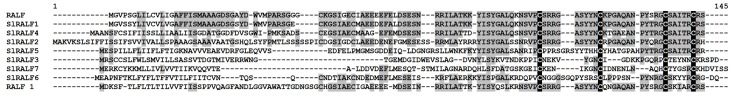
Multiple sequence alignment of *S. lycopersicum* RALF precursors and RALF from *Nicotiana tomentosiformis* (XP_00962090.1), RALF 1 from *A. thaliana* (NP_171789.1). Cysteine residues are shaded black, and identical amino acids are shaded gray.

Seven sequences encoding SlRALF precursors were discovered in tomato transcriptomes (Figure 11, Supplementary Materials Table S3). Six SlRALFs (SlRALF1−6) showed 100% sequence identity to RALFs and RALF-like peptides of *S. lycopersicum*. SlRALF7 was 94.9% identical to the hypothetical protein EJD97_021215 from *S. chilense*; however, it was annotated as rapid alkalinization factor by InterProScan [21]. All discovered sequences had characteristic features of RALFs; among them, there were four conserved cysteines. Five of seven sequences had a dibasic site for proteolytic cleavage by the processing protease, which is involved in coordinating the immune response in *A. thaliana* [41,42,43]. All but one polypeptide possessed a conserved motif YI/LSY in the N-terminal region of the molecule shown to be engaged in receptor binding [44]. The motif RCRR in the C-terminal region is less conserved.

Expression analysis showed that five RALFs were responsive to experimental treatments (Figure 2, Supplementary Materials Table S4). These peptides differed in expression levels: SlRALF2, SlRALF3, and SlRALF7 were moderately expressed, while SlRALF5 and 6 were weakly expressed under experimental conditions. SlRALF3, 6, and 7 were down-regulated by infection at 2 dpi, while SlRALF3 and 7 were also down-regulated at 4 dpi (Supplementary Materials Table S5). SlRALF 5 and 6 were up-regulated by the elicitors and infection of the elicitor-pretreated plants. SlRALF2, 5, 6, and 7 were up-regulated in IR-displaying plants compared to infected non-pretreated plants (Supplementary Materials Table S6). The expression level of the remaining RALFs remained unchanged upon infection and/or elicitor treatment.

#### 2.3.8. MEG (Maternally Expressed Gene)

*MEG1* is specifically expressed in the basal layer of maize endosperm transport cells [45]. Related genes were discovered in other cereals. In maize, the MEG1 precursor contains a signal peptide and a mature peptide with eight cysteine residues arranged in the following motif: CX{4,9}CX{4,12}CXCCX{4,10}CX{6,12}CX{3}C. The role of the MEG1 peptide is not fully understood. It is supposed to act as a structural or defense protein. The role in regulation of nutrient transport from the maternal cells to the developing embryo is also discussed.

**Figure 12:**
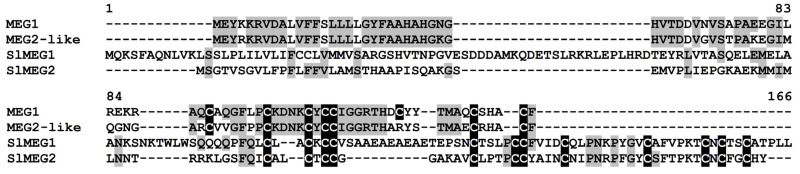
Multiple sequence alignment of *S. lycopersicum* MEG precursors and *Zea mays* MEG1 (XP_008652138.1), MEG2-like (XP_020397032.1). Cysteine residues are shaded black, and identical amino acids are shaded gray.

In tomato transcriptomes, two sequences named SlMEG1 and SlMEG2 possessing another cysteine motif CX{2}CXCCX{4,16}CX{3,4}CCX{4}CX{5,10}CX{6}CXCX{2,10}C characteristic of MEG-like polypeptides were discovered. They were annotated by BLAST as uncharacterized proteins of *S. lycopersicum* (Supplementary Materials Table S3). Despite a similar cysteine motif, they showed low sequence similarity with each other (Figure 12). They also differed considerably in the size of the molecule and predicted isoelectric point (PI) values.

The expression levels of both MEG genes were similar. However, expression profiling revealed differences in response to infection and elicitors. SlMEG2 was down-regulated by infection both at 2 and 4 dpi, while SlMEG1 was down-regulated by the elicitors (Figure 2, Supplementary Materials Tables S4 and S5).

#### 2.3.9. Ole e 1 and Ole e 6

Ole e 1 and Ole e 6 are pollen allergens of *Olea europaea* L. [46]. Ole e 1 is a polypeptide whose molecular weight is about 20 kDa, so it is a low-molecular-weight protein rather than peptide. It contains six conserved cysteine residues and shows sequence similarity to pollen proteins of other species (maize, rice, arabidopsis, and birch) [47].

Ole e 6 is an acidic cysteine-rich polypeptide of approximately 50 residues containing six cysteine residues. The three-dimensional structure of the polypeptide includes two antiparallel α-helices connected by a short loop and a long unstructured C-terminal tail [48]. The biological role of Ole e 1 and Ole e 6 remains unknown.

**Figure 13:**
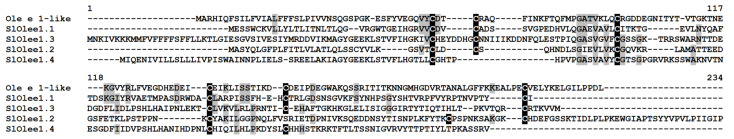
Multiple sequence alignment of *S. lycopersicum* Ole e 1 precursors and *O. europaea* Ole e 1-like (XP_022872526.1). Cysteine residues are shaded black, and identical amino acids are shaded gray.

Four sequences of precursors of Ole e 1-like polypeptides with low sequence similarity were discovered in tomato transcriptomes. SlOlee1.1 and 1.3 had a typical 6-Cys motif, while SlOlee1.4 had only four conserved cysteine residues, and in SlOlee1.2, the spacings between the fourth, the fifth, and the sixth cysteine residues differed from the canonical motif (Figure 13). Only one polypeptide was annotated by BLAST as pollen protein Ole e 1-like of *S. lycopersicum*, while the remaining polypeptides were annotated as uncharacterized proteins of *S. lycopersicum* (Supplementary Materials Table S3). However, all discovered precursor proteins were annotated as Ole e 1-like proteins by InterProScan [21].

Of Ole e 1 genes, the SlOlee1.1 expression level remained unchanged under the experimental treatments (Supplementary Materials Table S4). SlOlee1.2 was expressed at a moderate level and was down-regulated by infection at 4 dpi, elicitor treatment, and in IR-displaying plants (Figure 2, Supplementary Materials Table S5). Although the expression levels of SlOlee1.3 and 1.4 were low in all tomato transcriptomes, SlOlee1.3 was up-regulated by infection at 2 dpi, and SlOlee1.4 was up-regulated by infection at both infection stages and in IR-displaying plants (Figure 2, Supplementary Materials Table S5).

**Figure 14:**
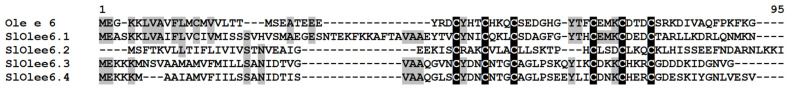
Multiple sequence alignment of *S. lycopersicum* Ole e 6 precursors and *C. baccatum* Ole e 6 (PHT46953.1). Cysteine residues are shaded black, and identical amino acids are shaded gray.

Four polypeptides named SlOlee6.1−SlOlee6.4 with a conserved 6-Cys motif were discovered in tomato transcriptomes (Figure 14, Supplementary Materials Table S3). Only SlOlee6.1 was annotated both by BLAST and InterProScan as pollen allergen Ole e 6-like protein of *S. lycopersicum*, showing high sequence similarity with Ole e 6 protein of *Capsicum baccatum* (Supplementary Materials Table S3). The remaining polypeptides showed 94.6–98.7% sequence identity to uncharacterized proteins of Solanaceae plants. The expression level of SlOlee6.1, SlOlee6.3, and SlOlee6.4 genes did not change under the experimental conditions. Conversely, the SlOlee6.1 gene was suppressed by the infection and elicitors; however, it was up-regulated in IR-expressing plants relative to the infected plants (Figure 2, Supplementary Materials Tables S5 and S6).

#### 2.3.10. EPF-Like Peptides

Epidermal patterning factor (EPF) peptides consist of 45–76 amino acid residues including six or eight cysteine residues [7]. Similarly to most families of CRPs, EPF peptides are represented by groups of related peptides. The 3D structure of *A. thaliana* EPF9 (stomatogen) is represented by two antiparallel β-strands connected by a loop. Molecular modeling showed that in EPF-1 and 2-like peptides, a disulfide bond is present in the loop, which is missing from the EPF-9-like peptides [49]. EPF peptides are synthesized as preproteins. These peptides are involved in stomatal development [50]. In transgenic plants overexpressing EPF genes, either stomatal development was inhibited or stomatal density was increased.

**Figure 15:**
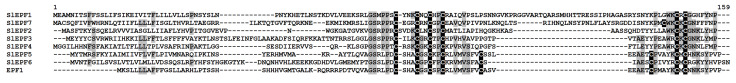
Multiple sequence alignment of *S. lycopersicum* EPF precursors and *A. thaliana* EPF1 [51]. Cysteine residues are shaded black, and identical amino acids are shaded gray.

In tomato transcriptomes, we discovered seven sequences encoding precursors of EPF peptides (Supplementary Materials Table S3). They showed low sequence similarity to each other except for the conserved cysteine residues (Figure 15). All of them were annotated as epidermal patterning factor-like proteins; two of them were new, showing 99.3% sequence similarity to the EPF-like protein from *S. lycopersicum* (SlEPF1) and 97.4% sequence similarity to EPF-like protein from *S. tuberosum* (SlEPF6).

The expression level of the SlEPF1 gene was higher than that of other DEGs of the family (Figure 2), while that of SlEPF6 and SlEPF7 was the lowest. Transcription profiling showed that two genes, SlEPF1 and SlEPF5, were down-regulated by infection with *F. oxysporum*; the latter and the SlEPF3 gene were also down-regulated by the elicitors (Supplementary Materials Tables S4 and S5). Conversely, SlEPF7 was up-regulated under all treatments, and SlEPF6 was up-regulated by the elicitors and in IR-displaying plants (Supplementary Materials Tables S4 and S5).

#### 2.3.11. PR-1 Proteins

PR-proteins comprise structurally diverse groups of polypeptides induced by different biotic and abiotic stressful factors [52]. PR-1 family members are low-molecular weight polypeptides with six cysteine residues, which are abundantly synthesized in plants in response to pathogen attack. Although they were discovered several decades ago and used as markers of SAR (systemic acquired resistance), their role in plant defense remains largely unknown. Recent studies discovered new properties of these proteins, shedding light into their involvement in host–pathogen interactions: they possess an embedded defense signaling peptide, exhibit sterol-binding activity, and serve as targets for plant pathogens [53].

**Figure 16:**
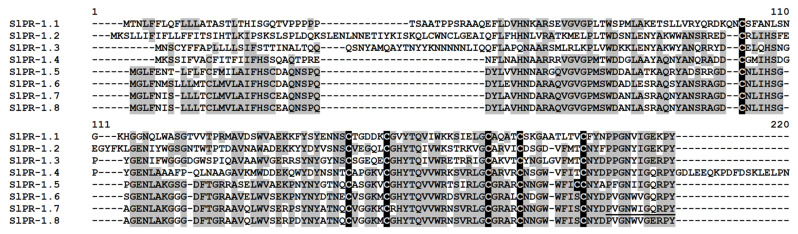
Multiple sequence alignment of *S. lycopersicum* PR-1 precursors. Cysteine residues are shaded black, and identical amino acids are shaded gray. The mature CAPE1 peptide sequence is underlined.

Eight sequences encoding precursors of PR-1 family proteins were discovered in tomato transcriptomes (Figure 16, Supplementary Materials Table S3). PR-1 protein precursors consist of a signal peptide and a mature peptide region of 135 to 177 amino acid residues long, including six conserved cysteine residues. All of them were annotated as PR-proteins of the PR-1 family by BLAST and as cysteine-rich secretory protein-related by InterProScan (Supplementary Materials Table S3). Five polypeptides are basic, and three polypeptides are acidic. Acidic PR proteins were shown to be mostly secreted polypeptides, while basic PR proteins were usually located in the vacuoles [54,55]. Polypeptides SlPR-1.5−SlPR-1.8 showed high sequence similarity, while the amino acid sequences of SlPR-1.1−SlPR-1.4 were more diverse (Figure 16). Two main clades comprising related polypeptides are seen on the phylogenetic tree (Supplementary Materials Table S3).

Expression profiling showed that SlPR-1 protein genes differed in expression levels (Figure 2, Supplementary Materials Table S4). The expression level of SlPR-1.7 was the highest among all discovered cysteine-rich polypeptides (Figure 2). However, it was not changed by the infection or the elicitors, but it was down-regulated in IR-displaying plants both against control and infected plants (Supplementary Materials Tables S5 and S6). SlPR-1.8 and SlPR-1.5 genes were also strongly expressed in tomato transcriptomes. However, they behaved differently. SlPR-1.8 was up-regulated by infection with *F. oxysporum* relative to control plants and down-regulated in IR-displaying plants relative to infected plants (Supplementary Materials Tables S4 and S6). SlPR-1.5 was suppressed by infection with the fungus, and its expression level did not change significantly in IR-expressing tomato plants. The expression levels of SlPR-1.1 and SlPR-1.6 genes were lower than those of PR-protein genes described above. However, SlPR-1.1 was up-regulated in IR-displaying plants relative to control and infected plants (Supplementary Materials Table S4). In contrast, the SlPR-1.6 gene was down-regulated in IR-expressing plants. The expression level of the remaining three PR-protein genes was low; however, SlPR-1.4 was up-regulated by all treatments, SlPR-1.2 was up-regulated in IR-displaying plants relative to control, infected, and induced plants. The SlPR-1.3 gene was also up-regulated in IR-expressing plants (Supplementary Materials Table S4).

#### 2.3.12. PR-4 Proteins

The proteins of the PR-4 family are represented by polypeptides, whose molecular weights are in the range of 15–20 kDa [56]. They are produced as precursors, which have a signal sequence and a mature domain of ≈120 amino acid residues long containing six cysteine residues, which is called the PR-4 domain. The proteins of the family are divided into two main classes. Class II proteins include tobacco PR-4a, which is induced by TMV infection, a barley seed protein called barwin [57], and wheatwin 1-4 isolated from wheat [58,59]. The genes of wheat PR-4 proteins have been cloned and shown to be selectively induced by pathogens, inducers of SAR, and wounding [60]. PR-4 proteins were also demonstrated to be involved in abiotic stress response [56]. The three-dimensional structure of barwin was solved by NMR spectroscopy [61]. The structure of the protein is formed by two β-layers, each of which consists of four antiparallel β-strands. Antifungal activity in vitro was confirmed for several proteins of the PR-4 family [62,63].

**Figure 17:**
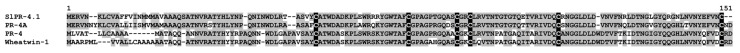
Multiple sequence alignment of *S. lycopersicum* PR-4 precursor and *Nicotiana tabacum* PR-4A (P29062.1), *Hordeum vulgare* pathogenesis-related protein 4 (PR-4) (KAE8805200.1), and *T. aestivum* wheatwin-1 (O64392.1). Cysteine residues are shaded black, and identical amino acids are shaded gray.

One polypeptide of the PR-4 family was predicted in the tomato transcriptome data (Figure 17, Supplementary Materials Table S3). It was weakly expressed in all four transcriptomes. No changes in the expression levels of this protein gene were observed in infected and elicitor-treated plants. However, in IR-expressing plants, it was down-regulated (Figure 2, Supplementary Materials Table S4).

### 2.4. Validation of RNA-Seq Data by RT-PCR and qRT-PCR

Expression of the SlHev1 gene was confirmed by RT-PCR. For this purpose, total tomato RNA was reverse transcribed, and PCR with specific primers (Supplementary Materials Table S7) was carried out. The obtained sequence was identical to that produced by Illumina sequencing. Thus, the sequence of SlHev1 gene was confirmed.

**Figure 18:**
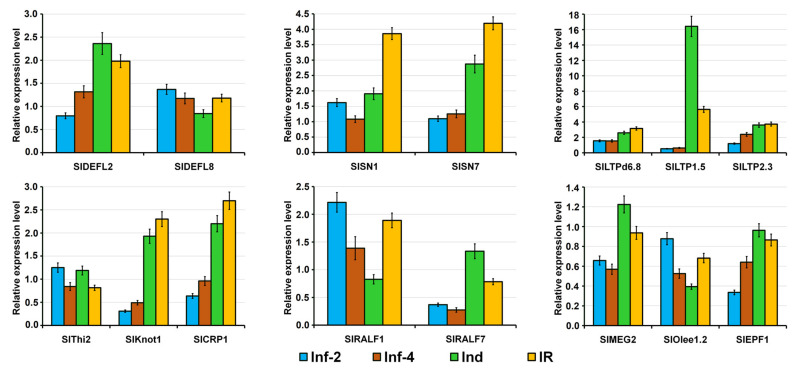
qRT-PCR validation of expression levels for selected *S. lycopersicum* CRP genes. Relative expression values were normalized using the EF1-α gene as internal control and standardized relative to the control values. Analyses were accomplished in triplicate. Bars represent mean ± standard deviation (SD).

To prove the expression profiles of 15 CRP genes generated by RNA-seq, we used qRT-PCR. The EF1-α gene was used as an internal control. Our qRT-PCR results confirmed that the expression patterns of all selected genes were consistent with the RNA-seq data (Figure 18).

## 3. Discussion

The strategy of mounting resistance in plants by biogenic elicitors represents an alternative to fungicide-based disease control of great economic potential [5]. Mycorrhizal fungi, as well as *Trichoderma* fungi and non-pathogenic *Fusarium* strains, can lead to induced systemic resistance in plants, protecting them against a wide range of pathogens [64,65]. However, efficient exploitation of bio-based resistance inducers requires extensive studies of their mode of action for a particular plant–elicitor–pathogen combination. Meanwhile, the mechanisms of induced resistance triggered by biogenic elicitors remain unexplored in Solanaceae species [66]. Of special importance is elucidation of the role of cysteine-rich peptides overrepresented in plant genomes in resistance mechanisms, whose involvement in induced immunity is only beginning to become clear [22,67]. The accumulating evidence suggests an essential role of peptides in plant’s physiology. Peptides are involved in plant growth, development, reproduction, symbiotic relationships, and stress response [68]. Some peptides, namely AMPs, directly inhibit the growth of pathogenic microorganisms [69], while others act as signaling molecules in an intercellular communication network [70].

Earlier, we demonstrated that the intracellular metabolites of *F. sambucinum* strain FS-94 effectively protect wheat from *F. oxysporum* infection by significantly up-regulating sets of genes encoding DEFLs and nsLTPs [22,67]. The aim of this study was to explore the role of different families of cysteine-rich peptides including not only AMPs but other CRP families in immune response of tomato plants to infection with *F. oxysporum* and treatment with FS-94 elicitors using RNA-seq. For the first time, the sets of CRP genes differentially expressed in tomato plants infected with the fungus, treated with *F. sambucinum* resistance-inducing elicitors, and infected plants after elicitor treatment were studied. CRP peptides and low-molecular weight defense proteins involved in defense response to infection and elicitors were identified.

**Figure 19:**
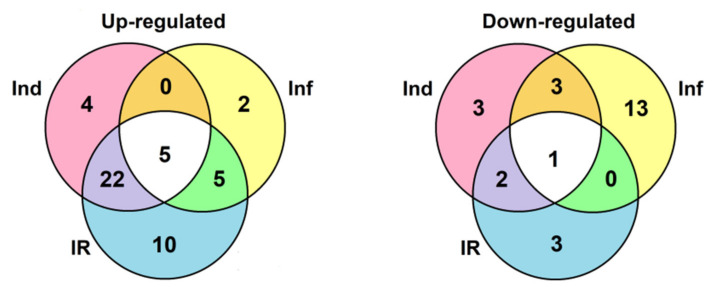
Venn diagram showing the number of CRP genes specifically up- or down-regulated compared to control in elicitor-treated (Ind), *F. oxysporum*-infected at 4 dpi (Inf), and IR-displaying *S. lycopersicum* plants as well as similarly expressed CRPs in all three transcriptomes. For up-regulated genes, expression fold change was ≥2, for down-regulated genes, it was ≤0.5.

A total of 106 sequences of cysteine-rich peptides and short proteins were predicted in tomato transcriptomes. All of them were synthesized as pre- or preproproteins. Note that in the tomato genome, 191 putative CRPs were discovered [16]. Thus, about one-half of the genes encoding CRPs were expressed in tomato plants under the experimental conditions used. All, except one, discovered sequences belong to known families of AMPs, regulatory peptides, and PR-proteins (Supplementary Materials Table S3). The most abundant were nsLTPs and snakins (Figure 2 and Figure 4). They comprise 53% of all predicted peptides. A total of 71 genes encoding cysteine-rich peptides and short proteins of the PR-1 and PR-4 families were differentially expressed in tomato leaves upon infection with *F. oxysporum*, treatment with the elicitors, and in IR-displaying plants (Figure 19, Supplementary Materials Table S4). Among tomato DEGs, nsLTPs and snakins also prevailed (Figure 4). Interestingly, not only AMPs and immune-related peptides (RALFs) were differentially expressed in tomato upon *F. oxysporum* infection and elicitor treatment. Some other CRP families, such as MEG, Ole e 1 and e 6, and EPF implicated in developmental processes changed expression, suggesting their involvement in the immune response in tomato (Figure 4, Supplementary Materials Table S4). However, the suppression of development-related mechanisms upon fungal infection cannot be ruled out.

**Figure 20:**
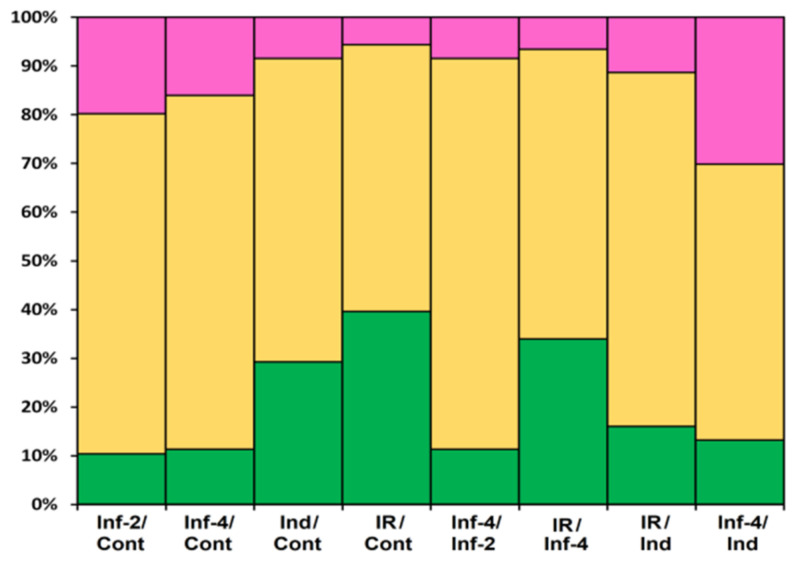
Differentially expressed CRP genes in *S. lycopersicum* transcriptomes (as a percentage of the total number of expressed CRP genes). Up-regulated genes (expression fold change ≥2) are colored green; down-regulated genes (expression fold change ≤0.5) are colored pink; genes whose expression level did not change are shown in yellow. The designations below the figure are as follows: Inf/Cont, infected at 2 and 4 dpi versus control; Inf-4/Inf-2, infected at 4 dpi versus infected at 2 dpi; Ind/Cont, elicitor-treated versus control; IR/Cont, IR-expressing versus control; IR/Ind, IR-expressing versus elicitor-treated; IR/Inf-4, IR-expressing versus infected at 4 dpi; Inf-4/Ind, infected at 4 dpi versus elicitor-treated.

The portion of DEGs upon *F. oxysporum* infection, *F. sambucinum* elicitor treatment, and infection of elicitor-treated plants was different, increasing in the row: Inf 2 dpi < Inf 4 dpi < Ind < IR (Figure 20). The portion of down-regulated genes decreased in the same order. Tomato plants responded to different treatments by modifying the expression of distinct overlapping sets of genes, some of which were up-regulated, while others were down-regulated (Figure 2, Supplementary Materials Table S4).

Infection with the fungus was studied at two time points, 2 and 4 dpi. At both stages of the infectious process, more genes were suppressed than up-regulated: 21 at 2 dpi and 17 at 4 dpi (Figure 20, Supplementary Materials Table S5). The prevalence of down-regulated CRP genes over up-regulated was also observed in our studies of *Stellaria media* transcriptomes upon infection with *F. oxysporum* [71]. In tomato, snakins, Type D nsLTP, and RALF genes were down-regulated both at 2 and 4 dpi, as well as single peptide genes belonging to other families, such as SlKnot1, SlMEG2, SlEPF5, and SlOlee6.2. Several genes were up-regulated at both time points, including AMPs (SlDEFL4 and SlHev1, Type G SlLTPs (g1.1, g2.6, g2.7), and Type X SlLTP (x2.1)) and non-AMPs (SlOlee1.4 and SlEPF7) (Supplementary Materials Table S5). In contrast, some CRP genes (SlLTPg2.4 and SlOlee1.3) were specifically up-regulated at 2 dpi, while others (SlLTP1.2, SlLTP2.3 and SlKnot2) were up-regulated only at 4 dpi. In addition to peptides, infection with *F. oxysporum* in tomato induced expression of the PR-1 family proteins: SlPR-1.4 at 2 dpi and SlPR-1.8 at 4 dpi (Supplementary Materials Table S5). Interestingly, another PR-1 family protein, SlPR-1.5, was down-regulated at 2 dpi. The up-regulation of some members of the CRP gene family and down-regulation of others suggests the diversification of functions within the peptide/protein families. The suppression of defense peptide/protein gene expression by the fungus may indicate inhibition of the plant’s immune response by the pathogen, while the up-regulation of peptide genes points to the activation of the defense reactions in response to pathogen invasion.

Treatment with the elicitors activated the expression of more genes than infection with *F. oxysporum* (Figure 20, Supplementary Materials Table S5). Moreover, in contrast to *F. oxysporum* infection, significantly more genes were up-regulated (31 versus 12) than down-regulated (Figure 20, Supplementary Materials Table S5). The majority of up-regulated genes belonged to the snakin and Type D nsLTP families. Expression level of snakin genes increased 2–10 times, and that of nsLTPs increased 2–26 times (Supplementary Materials Table S4). Two up-regulated nsLTP genes encoded SlLTPd2.2 and d2.3, which were annotated as putative lipid-transfer protein DIR1, and one up-regulated nsLTP gene encoded nsLTPg2.5 with an acidic PI, suggesting that it also belongs to DIR1-like nsLTPs (Supplementary Materials Table S3). It was established that DIR1 operates as a mobile signal or chaperone that is induced in locally infected leaves, which moves via phloem to establish SAR in distant leaves [72]. Thus, we suppose that SlLTPd2.2−d2.3 and SlLTPg2.5 exhibit not antimicrobial but signaling functions. Only 2 snakin and 2 nsLTP genes of AMP genes were down-regulated, including SlLTPg1.2 with acidic PI and a putative signaling role. Taking into account that the majority of genes up-regulated by the elicitors encode AMPs, we may conclude that *F. sambucinum* elicitors evoke potent antifungal response (Supplementary Materials Table S5). The activation of two genes encoding EPFs and the suppression of two others may be associated with elicitor-induced changes in stomata development.

Comparison of gene expression in IR-displaying and control plants revealed the up-regulation of 42 genes and down-regulation of six genes (Figure 19, Supplementary Materials Table S5). Similarly to elicitor-treated plants, among up-regulated genes, snakin and nsLTP genes prevailed. In addition to the genes activated by the elicitors and fungal infection, several novel genes were up-regulated in IR-expressing plants encoding SlSN1, SlSN9, SlLTPd3.1, SlLTPd6.5, SlLTPg2.4, Slknot1, SlPR-1.1, -1,2, and -1.3 (Supplementary Materials Table S5). These genes were primed by the elicitors, and they obviously contribute to the enhanced antimicrobial potential of the tomato plants pretreated with the elicitors. Four genes of the PR-1 family, markers of SAR, were up-regulated. Three PR-protein genes (SlPR-1.6, SlPR-1.7 and SlPR-4.1) were down-regulated. For SlPR-1.7, it was shown that the endogenous peptide CAPE1 derived from the C-terminal region of this protein serves as a signal in tomato response to pathogens [73]. Homologous peptides were found in other tomato PR-1 family proteins (Figure 16). We may speculate that up-regulated PR-1 genes generate CAPE1 homologues that positively regulate defense response in tomato, while down-regulated genes act as negative regulators of the immune response.

**Figure 21:**
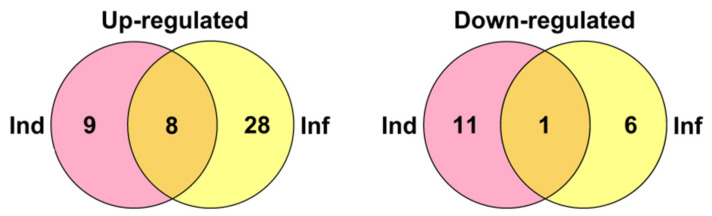
Venn diagram showing the number of CRP genes specifically up- or down-regulated in IR-displaying plants compared to elicitor-treated (Ind) and *F. oxysporum*-infected at 4 dpi (Inf) seedlings as well as similarly expressed genes in both transcriptomes. For up-regulated genes, expression fold change was ≥2, for down-regulated, it was ≤0.5.

Comparison of gene expression in IR-expressing plants with that of infected plants without elicitor treatment disclosed the up-regulation of as many as 36 genes, of which 14 and 9 belonged to nsLTPs and snakins, respectively (Figure 21, Supplementary Materials Table S6). Putative DIR1-like SlLTPd2.2, SlLTPd2.3, and SlLTPg2.9 were up-regulated. Up-regulated genes also included four RALFs, one DEFL, one thionin-like peptide, two knottin-like peptides, one Ole e 6, and three PR-1 protein genes. Only seven genes were down-regulated in IR-expressing plants compared to infected ones.

Thus, analysis of DEGs clearly demonstrates that snakins and nsLTPs mostly contribute to the response of tomato plants to *F. oxysporum* infection and treatment with *F. sambucinum* elicitors under our experimental conditions (Figure 4, Supplementary Materials Table S4). Infection with the fungus up-regulated several TypeG nsLTP genes and down-regulated snakin and Type D nsLTP genes. Conversely, treatment with the elicitors enhanced the expression of snakin and Type D nsLTP genes. Thus, the separation of Type D and Type G nsLTP subfamilies reflects not only structural differences but functional differentiation between these subfamilies as well. Types 1 and 2 nsLTPs were up-regulated both by the fungal infection and elicitors, pointing to their involvement in non-specific response to environmental changes. Other players in this non-specific response are SlHev1, SlKnot2, and SlEPF7, which were up-regulated at all treatments (Supplementary Materials Table S5).

In addition to revealing different sets of genes involved in plant response to *F. oxysporum* infection and elicitor treatment, transcriptome analysis allowed us to discover a new type of 10-Cys-containing hevein-like peptides. It deserves special attention that before our study, only three types of hevein-like peptides with a 10-Cys motif, which differ in the position of the fifth disulfide bond, were reported: EAFP1 [29], Ee-CBP [30], and WAMP-1 [31]. In tomato, we discovered a gene encoding a hevein-like peptide named SlHev1 with a novel, fourth type of cysteine arrangement in the mature peptide (Figure 7). This novel gene encodes a precursor protein containing a signal peptide, a mature peptide domain with 10 Cys residues and a short C-terminal prodomain, which was up-regulated both by *F. oxysporum* inoculation and *F. sambucinum* elicitor treatment that implicates its participation in non-specific defense response in tomato plants. The predicted SlHev1 peptide is basic and shows sequence similarity to other hevein-type peptides (Figure 7). In the tomato genome, a peptide homologue annotated as a hypothetical protein EJD97_021591 (TMW80309.1) of *S. chilense* with 96.6% similarity to SlHev1 was identified. Highly similar peptides were discovered in other plants of the Solanaceae family by database search (Figure 8). The conservation of sequences of SlHev1 homologues points to their crucial role in plant’s physiology. A similar conclusion was drawn when we analyzed sequence conservation among WAMP homologues in grasses [74]. Thus, the repertoire of plant AMPs has been expanded by a novel type of 10-Cys hevein-like peptides.

## 4. Materials and Methods

### 4.1. Biological Material

Seeds of *Solanum lycopersicum* L. (cultivar Belyi naliv) susceptible to *Fusarium* wilt were used in the experiments. The seeds were surface-sterilized, germinated, and sown in disinfected sand as described earlier [12].

*Fusarium sambicinum* strain FS-94 (non-pathogenic for wheat and tomato) and the pathogenic isolate of *F. oxysporum* f. sp. *lycopersici* F37 were obtained from the State Collection of Plant Pathogenic Microorganisms at the All-Russian Research Institute of Phytopathology (Moscow region, Russia).

### 4.2. Isolation of FS-94 Elicitors

The elicitor metabolites of *F. sambucinum* (strain FS-94) were isolated using the procedure described earlier [13,75]. In short, the elicitor-containing fraction was obtained by extraction of mycelium followed by purification of the extract by gel filtration on Sephadex G-50 and ultrafiltration. The protein-containing fraction was further purified by size-exclusion chromatography on Sephacryl S-400 (GE Healthcare, Boston, MA, USA) as described [75]. Protein concentration was determined using Bradford protein assay kit (Bio-Rad, Hercules, CA, USA).

### 4.3. Plant Protection Assay

Tomato plants grown to the stage of 4–5 true leaves from untreated surface-sterilized tomato seeds were carefully washed from the substrate and immersed for 48 h in an aqueous solution of the elicitors or in sterile distilled water (control) in 50% Knop’s solution so as to cover the roots and stems but not the leaves. Five seedlings for each treatment in 4 replicates were taken. After incubation, roots were washed twice with sterile distilled water (sdW). Tomato seeds were treated with different concentrations of the elicitors (200, 100, and 50 μg/mL) as described in [13]. In the experiments with seedlings, prior *to F. oxysporum* inoculation, the root tips of control and elicitor-treated young plants were cut by 1-2 mm, whereupon the seedlings were immersed in the suspension of conidia of the pathogenic F37 strain (final concentration of 1 × 10^6^ conidia/mL in 50% Knop’s solution) for 48 h. Some of the control seedlings were left uninfected (non-inoculated control).

Wilt symptoms were evaluated visually for each plant 5 and 10 days after the inoculation using a five-point scale [12], and the averaged disease score and the treatment efficacy (%) relative to control were calculated.

### 4.4. Experimental Design

For transcriptome analysis, tomato seedlings were grown in the disinfected sand to the stage of 5 true leaves, whereupon the stems with 4–5 leaves were cut from the roots. The seedlings were divided into 5 groups (4 seedlings per experimental variant). The setup of the experiment is summarized in Table 2. Seedlings of the first group (control) were incubated in Knop’s solution for 4 days. The second group of seedlings was incubated in the pathogenic *F. oxysporum* strain F37 spore suspension (1 × 10^6^ conidia/mL) for 2 days and then transferred to Knop’s solution and incubated for 2 more days. Seedlings of the third group (elicitor-treated) were kept for 2 days in the solution of FS-94 metabolites at a concentration of 200 μg/mL followed by incubation in Knop’s solution for 2 days. The fourth group of seedlings was incubated in Knop’s solution for 2 days and then in *F. oxysporum* spore suspension for 2 more days. The fifth group (elicitor-treated and inoculated) was treated with the elicitors for 2 days and then transferred to *F. oxysporum* spore suspension and incubated for 2 more days. Before use, the elicitor fraction and the Knop’s solution were sterilized using the Millipore membrane (0.22 μm). During the whole experiment, a constant volume of incubation solutions was maintained (25 mL per seedling). After incubation, the samples for RNA isolation were collected (90−100 mg of leaf tissue), whereupon they were frozen in liquid nitrogen and stored at −72 °C before use.

Thus, 5 plant samples (in two biological replicates) for RNA isolation were obtained from tomato seedlings: (1) Control (Cont), (2) Elicitor-treated (Ind), (3) Inoculated with *F. oxysporum* (at the first day of the experiment, Inf-4), (4) Inoculated with *F. oxysporum* (at the third day of the experiment, Inf-2), (5) Elicitor-treated and inoculated (IR).

### 4.5. RNA Isolation

Total RNA was isolated from 100 mg of plant material using the ExtractRNA kit (Evrogen, Moscow, Russia) according to the manufacturer’s protocol. The quality of RNA samples was checked with NanoDrop 2000 (Thermo Fisher Scientific, Wilmington, DE, USA) and Agilent 2100 Bioanalyzer (Agilent, Santa Clara, CA, USA). One half of each RNA sample was used for production of ten cDNA libraries for Illumina HiSeq4000 sequencing; the remaining half was used for qRT-PCR validation.

### 4.6. Library Construction and NGS

The mRNA isolation from the total RNA of ten *S. lycopersicum* samples using RNA purification beads followed by fragmentation and priming for cDNA synthesis was performed as recommended by the manufacturer (Illumina, San Diego, CA, USA) and described earlier [67]. Double-stranded cDNA synthesis was carried out with the SuperScript Double-Stranded cDNA Synthesis kit (Invitrogen, Carlsbad, CA, USA). For further purification, Agencourt AMPure XP beads (Beckman Coulter, Indianapolis, IN, USA) were used. End repairing and 3′-ends adenylation were accomplished following the RNA adapters ligation. Upon enrichment of the DNA fragments library, templates were validated using Agilent 2100 Bioanalyzer (Agilent, Santa Clara, CA, USA). Clonal clusters were produced from DNA library templates with the TruSeq PE Cluster Kit v2 and cBot automated system (Illumina, San Diego, CA, USA). Clusters obtained were used to perform paired-end runs on an Illumina HiSeq4000 instrument (101 cycles from each fragment end) using HiSeq 4000 sequencing kit version 1 (Illumina, San Diego, CA, USA). FASTQ files were obtained with bcl2fastq v2.17.1.14 Conversion Software (Illumina, San Diego, CA, USA).

### 4.7. Sequencing Data Analysis

Sequence reads from each sample were trimmed using trimmomatic software version 0.30 with parameters «ILLUMINACLIP: adapters.fa:2:30:10 LEADING:20 TRAILING:20 SLIDINGWINDOW:4:20 MINLEN:40» [76]. Trimmed reads from each sample were combined. Combined reads were assembled using rnaSPAdes software version 3.14.1 with default parameters [14]. Assembled transcripts were annotated with TransDecoder version 5.5.0 software (https://github.com/TransDecoder/TransDecoder/wiki, accessed on 13 april 2020). Submodule TransDecoder.LongOrfs was used with default parameters. Submodule TransDecoder.Predict was employed with parameter «--single_best_only».

### 4.8. Identification of CRP Precursors in Tomato Transcriptomes

The pipeline for the identification of putative CRPs was written in Perl. The core of the algorithm was based on regular expressions. The pipeline consisted of several steps.

First, nucleotide genomic sequences were translated into protein sequences by 6 reading frames using transeq from the EMBOSS package (http://emboss.open-bio.org/). Then, it searched for strings that fit the regular expression in protein sequences in FASTA format. The regular expressions had the following structure: MZ..Z{C}m{X}n{C}l{X}k..*, where MZ..Z is a signal peptide; M, methionine; Z, any amino acid; C, cysteine; X, any amino acid residue except cysteine; m, n, l, k = 1, 2, 3 . . . ; * is a stop codon. Cysteine structures matched conservative motifs of different CRP families: defensins, snakins, hevein-like peptides, non-specific lipid transfer proteins, knottin-like peptides, thionins, MEG peptides, Ole e 1 and 6, RALFs, PR-1, and PR-4 proteins. All the structures for the motifs were previously described by Silverstein et al. [8]. At the end of this stage, amino acid sequences of the putative precursors were defined.

At the second stage, putative CRP precursors were sorted to form the non-redundant list of sequences. Next, they were processed using SignalP to predict signal peptides [77]. Sequences without signal peptides were discarded from the analysis.

Finally, all identified putative CRPs were annotated using online version of BLAST and checked using the CAMPr3 program to predict if they belong to antimicrobial peptides [78]. For domain identification, the InterProScan program was used [21]. Molecular weight and PI for each putative CRP were calculated by ProtParam [79]. The C-terminal GPI-anchored signals of LTPs were predicted by the big-PI PPlant Predictor program [80]. Three-dimensional (3D) structure modeling was carried out using SWISS-MODEL [35]. All alignments and phylogenetic tree construction were carried out with the Vector NTI Advance 9 software. Sequence Logo plots of aligned sequences were generated using WebLogo [81].

The corresponding nucleotide sequences of the putative peptides were detected by a specific script written in Perl. It obtains the coordinates of the identified putative CRP precursors in the translated protein sequences, back-translates them into the coordinates in the original genomic sequences depending on the reading frame, and then gets the final nucleotide sequences by these coordinates.

### 4.9. Differential Gene Expression Analysis

Trimmed reads from 10 libraries were mapped on *S. lycopersicum* genome version SL3.0 produced by combining all libraries using STAR version 2.5.1b software with parameter «--quantMode GeneCounts» [82]. Read counts were used for estimating differentially expressed genes with DeSeq2 software version 1.30.1 [83].

Expression values for individual coding sequences were calculated as Counts per Million Mapped Reads (CPM). Minimal expression threshold was defined as 0.3. Differentially expressed genes were those with an expression fold change ≥2 (up-regulation) or ≤0.5 (down-regulation) and FDR ≤0.5.

CRP gene expression patterns were represented by heat maps (R package gplots v3.0.1).

### 4.10. RT-PCR Validation of SlHev1 Expression

One microgram of total RNA obtained by combining RNA preparations from all samples were used for the rapid amplification of cDNA ends using the Mint kit (Evrogen, Moscow, Russia) according to the manufacturer’s instructions. The amplified cDNA coding SlHev1 was synthesized using gene-specific primers (Supplementary Materials Table S4) constructed using the Beacon Designer 4.0 program and high-fidelity Tersus DNA polymerase (Evrogen, Moscow, Russia). PCR conditions were as follows: initial denaturation step at 94 °C for 2 min followed by 35 cycles of denaturation at 94 °C for 30 s, primer annealing at 58 °C for 30 s, and primer extension at 72 °C for 30 s, with the final extension of 5 min at 72 °C. The amplified fragment was separated by agarose gel electrophoresis and isolated from the gel with the Cleanup Standard kit (Evrogen, Moscow, Russia). The PCR fragment was cloned into the pAL2-T vector (Evrogen, Moscow, Russia). The construct obtained was sequenced on an ABI PRISM 3730 instrument (Applied Biosystems, Foster City, CA, USA).

### 4.11. Real-Time PCR Analysis

To confirm the expression levels of 15 selected peptide genes obtained by RNA-seq, qRT-PCR was employed. One microgram of RNA from each biological replicate of the same sample was pooled and used for cDNA synthesis. The list of primers used in PCR is shown in Supplementary Materials Table S7. qRT-PCR was carried out with the qPCRmix-HS SYBR+HighROX kit (Eurogen, Moscow, Russia) in 20 μL reaction volume according to the manufacturer’s protocol on a DTX96™ Real-Time System (DNA-technology, Moscow, Russia). PCR conditions were as follows: initial denaturation step at 94 °C for 2 min followed by 40 cycles of denaturation at 94 °C for 30 s, primer annealing at 59–62 °C for 30 s, and primer extension at 72 °C for 30 s, with the final extension of 5 min at 72 °C. Candidate gene expression levels were normalized using the EF1-α (*Solanum lycopersicum* elongation factor 1-alpha) gene (LOC544045) as endogenous control. Each experiment was run in three technical replicates. The relative abundance of transcripts was estimated using the 2^−∆∆C^_T_ method [84].

The PCR amplification specificities of genes were confirmed by sequencing the PCR fragment. The results were presented as the mean ± standard deviation (SD).

## 5. Conclusions

In this work, using whole-genome transcriptome sequencing, we explored the repertoire and role of CRPs in tomato response to *F. oxysporum* infection and elicitors from *F. sambucinum* FS-94. We revealed 106 putative CRP transcripts and discovered a novel type of a 10-Cys hevein-like AMP named SlHev1 with a cysteine motif distinct from all known motifs for this AMP family. We showed that *F. oxysporum* and *F. sambucinum* elicitors changed the expression of different sets of CRP genes. The fungus suppressed the plant immune system by down-regulating CRP genes, among which AMPs (nsLTPs and snakins) prevailed. Still, some AMPs and non-AMPs, such as SlDEFL4 and SlHev1 and Type G and X nsLTPs, were up-regulated by the infection, pointing to activation of the defense response triggered by the fungus. The elicitors stimulated the immune response in tomato by significantly up-regulating a large number of defense-related CRPs. Among up-regulated genes, again, AMP genes prevailed; however, genes for signaling peptides DIR1-like nsLTPs, RALFs, and possibly CAPEs were also activated. Studies of the repertoire of CRPs predicted in the tomato plants displaying IR showed that the IR state was accompanied by expression of a plethora of CRPs belonging to different AMP families; among them were unique peptides that were not induced by the elicitors or the fungus alone (primed by the elicitors). Our study clearly demonstrates that snakins and nsLTPs mostly contribute to the response of tomato plants to *F. oxysporum* infection and treatment with *F. sambucinum* elicitors. The IR state in tomato triggered by the elicitors is associated with the expression of a large number of CRP genes involved in direct defense or signaling. We also demonstrated the involvement of CRP genes previously unrelated to defense in the immune response in tomato. Further studies will clarify the exact role of these CRPs in the molecular mechanisms involved.

## Figures and Tables

**Table 1:** Protective effect of FS-94 elicitors against Fusarium wilt of tomato.

Elicitor Fraction ^1^, µg/mL	Efficacy of Seedling Protection, % Relative to Control (M ± SE) ^2^	
	5 dpi	10 dpi
	Seed soaking	
200	76.4 ± 4.05	57.7 ± 2.43
100	58.5 ± 1.16	52.6 ± 2.13
50	46.5 ± 0.33	37.5 ± 0.96
	Root immersion	
200	74.0 ± 8.14	53.2 ± 3.86

^1^ Combined protein-containing fractions of the purified mycelial extract, which were isolated by size-exclusion chromatography on Sephacryl S-400 (see Materials and Methods). ^2^ Values of means (M) and standard errors (SE) are indicated. Differences from control (non-preteated and inoculated seedlings) are significant at *p* ≤ 0.05.

**Table 2:** Scheme of the experiment.

№	Variant	Incubation with FS-94 Elicitors	Inoculation with F37 Conidia
1	Non-inoculated control (Cont)	0 h	Without inoculation
2	Inoculation with *F. oxysporum* (Inf-4)	0 h	Immediate for 48 h
3	Treatment with elicitors (Ind)	Immediate for 48 h	Without inoculation
4	Inoculation with *F.oxysporum* (Inf-2)	0 h	After 48 h for 48 h
5	Treatment with elicitors followed by inoculation with *F. oxysporum* (IR)	48 h	After 48 h for 48 h

## Data Availability

Sequencing data were deposited in NCBI at BioProject accession number PRJNA724024.

## Supplementary Materials

The following are available online at https://www.mdpi.com/article/10.3390/ijms22115741/s1, Table S1: Reads statistics, Table S2: Assembly statistics, Table S3: *S. lycopersicum* CRPs, Table S4: Expression patterns of *S. lycopersicum* CRP genes, Table S5: CRP genes responsive to *F. sambucinum* elicitors, *F. oxysporum* infection and to *F. oxysporum* infection after elicitor treatment, Table S6: Up- and down-regulated CRP genes in IR-expressing *S. lycopersicum* plants, Table S7: List of primers for RT-PCR validation.
