# Supplementary material for: Molecular Insights into the Role of Cysteine-Rich Peptides in Induced Resistance to Fusarium oxysporum Infection in Tomato Based on Transcriptome Profiling
Source: Int J Mol Sci. 2021 May 27;22(11):5741. doi: 10.3390/ijms22115741 (PMC8198727; doi:10.3390/ijms22115741)
Supplement: Supplementary file 1 [file ijms-22-05741-s001.zip › Table S1.pdf]

Table S1. Reads statistics.

Sample	Number of raw reads	Number of trimmed reads	Number of uniquely mapped reads
Cont (a)	44368206	40463022	36396638
Cont (b)	80155548	72350380	66873112
Inf-4 (a)	60111090	54870442	51991574
Inf-4 (b)	78014612	71970474	66674266
Ind (a)	52592046	48434626	46017998
Ind (b)	59905032	54606326	50761996
Inf-2 (a)	63474308	58424784	53911254
Inf-2 (b)	64401390	59064290	54421814
IR (a)	148867186	137637508	131677132
IR (b)	123715738	114429278	105805514

Note: (a) and (b) − sample replicate.
