# Supplementary material for: Molecular Insights into the Role of Cysteine-Rich Peptides in Induced Resistance to Fusarium oxysporum Infection in Tomato Based on Transcriptome Profiling
Source: Int J Mol Sci. 2021 May 27;22(11):5741. doi: 10.3390/ijms22115741 (PMC8198727; doi:10.3390/ijms22115741)
Supplement: Supplementary file 1 [file ijms-22-05741-s001.zip › Table S2.pdf]

Table S2. Assembly statistics.

Parameters	Number of raw assembled contigs	Number of CDS
Number of sequences	137158	45095
N50, bp	2190	1341
Median length, bp	382	798
Average length, bp	1000,48	1036,39
Maximal length, bp	20864	15315
