# Supplementary material for: Molecular Insights into the Role of Cysteine-Rich Peptides in Induced Resistance to Fusarium oxysporum Infection in Tomato Based on Transcriptome Profiling
Source: Int J Mol Sci. 2021 May 27;22(11):5741. doi: 10.3390/ijms22115741 (PMC8198727; doi:10.3390/ijms22115741)
Supplement: Supplementary file 1 [file ijms-22-05741-s001.zip › Table S5.pdf]

Table S5. CRP genes responsive to *F. sambucinum* elicitors, *F. oxysporum* infection and to *F. oxysporum* infection after elicitor treatment [1]

Up-regulated				Down-regulated			
**Inf-2/Cont [2]**	**Inf-4/Cont [2]**	**Ind/Cont [3]**	**IR/Cont [4]**	**Inf-2/Cont [2]**	**Inf-4/Cont [2]**	**Ind/Cont [3]**	**IR/Cont [4]**
SlDEFL4	SlDEFL4	SlDEFL1	SlDEFL1				
		SlDEFL2	SlDEFL2				
		SlSN3	SlSN1	SlSN3	SlSN2	SlSN10	
		SlSN4	SlSN3	SlSN4	SlSN3	SlSN11	
		SlSN5	SlSN4	SlSN5	SlSN5		
		SlSN6	SlSN5	SlSN6	SlSN9		
		SlSN7	SlSN6	SlSN9	SlSN11		
		SlSN8	SlSN7	SlSN11			
			SlSN8				
			SlSN9				
		SlThi3	SlThi3				
SlLTPg1.1	SlLTP1.2	SlLTP1.1	SlLTP1.1	SlLTP1.5	SlLTPd6.1	SlLTP1.4	SlLTP1.4
SlLTPg2.4	SlLTP2.3	SlLTP1.2	SlLTP1.2	SlLTPd6.2	SlLTPd6.2	SlLTPg1.2	
SlLTPg2.6	SlLTPg1.1	SlLTP1.5	SlLTP1.5	SlLTPd6.3	SlLTPd6.3		
SlLTPg2.7	SlLTPg2.6	SlLTP2.1	SlLTP2.3	SlLTPd6.9	SlLTPd6.6		
SlLTPx2.1	SlLTPg2.7	SlLTP2.3	SlLTPd2.2	SlLTPd6.10	SlLTPd6.9		
	SlLTPx2.1	SlLTPd2.2	SlLTPd2.3	SlLTPg2.2			
		SlLTPd2.3	SlLTPd3.1				
		SlLTPd6.1	SlLTPd6.1				
		SlLTPd6.2	SlLTPd6.5				
		SlLTPd6.3	SlLTPd6.8				
		SlLTPd6.6	SlLTPd6.9				
		SlLTPd6.8	SlLTPd6.10				
		SlLTPd6.9	SlLTPg1.1				
		SlLTPd6.10	SlLTPg2.4				
		SlLTPg2.5	SlLTPg2.5				
			SlLTPg2.6				

			SlLTPg2.7				
			SlLTPx2.1				
SlHev1	SlHev1	SlHev1	SlHev1				
			SlKnot1	SlKnot1	SlKnot1		
	SlKnot2	SlKnot2	SlKnot2				
		SlRALF5	SlRALF5	SlRALF3	SlRALF3		
		SlRALF6	SlRALF6	SlRALF6			
				SlRALF7	SlRALF7		
				SlMEG2	SlMEG2	SlMEG1	
SlOlee1.3					SlOlee1.2	SlOlee1.2	SlOlee1.2
SlOlee1.4	SlOlee1.4		SlOlee1.4				
				SlOlee6.2	SlOlee6.2	SlOlee6.2	
		SlEPF6	SlEPF6	SlEPF1		SlEPF3	SlEPF3
SlEPF7	SlEPF7	SlEPF7	SlEPF7	SlEPF5	SlEPF5	SlEPF5	
			SlCRP1				
	SlPR-1.8		SlPR-1.1	SlPR-1.5			SlPR-1.6
			SlPR-1.2				SlPR-1.7
			SlPR-1.3				
SlPR-1.4		SlPR-1.4	SlPR-1.4				
							SlPR-4.1

[1] Differentially expressed genes are those with an expression fold change ≥2 (up-regulation) or ≤0.5 (down-regulation). [2] Genes responsive to *F. oxysporum* infection (Inf) at 2 and 4 dpi compared to control plants (Cont). [3] Genes responsive to *F. sambucinum* elicitors (Ind) compared to control plants (Cont). [4] Genes responsive to *F. oxysporum* infection after elicitor treatment (in IR-expressing plants) compared to control plants (Cont). Genes up-regulated in both Inf variants are highlighted orange (or light yellow in case genes up-regulated in all 4 variants). Genes up-regulated in all 3 variants (Inf-4, Ind, IR) are highlighted yellow. Genes up-regulated only in IR-expressing plants (primed by the elicitors) are highlighted blue. Genes down-regulated in both Inf variants are highlighted purple. Genes down-regulated in all 3 variants (Inf-4, Ind, IR) are highlighted green.
