# Supplementary material for: Molecular Insights into the Role of Cysteine-Rich Peptides in Induced Resistance to Fusarium oxysporum Infection in Tomato Based on Transcriptome Profiling
Source: Int J Mol Sci. 2021 May 27;22(11):5741. doi: 10.3390/ijms22115741 (PMC8198727; doi:10.3390/ijms22115741)
Supplement: Supplementary file 1 [file ijms-22-05741-s001.zip › Table S6.pdf]

Table S6. Up- and down-regulated CRP genes in IR-expressing *S. lycopersicum* plants [1]

Up-regulated		Down-regulated	
**IR/Ind [2]**	**IR/Inf [3]**	**IR/Ind [2]**	**IR/Inf [3]**
	SlDEFL1		SlDEFL4
SlSN1	SlSN1	SlSN3	
SlSN2	SlSN2	SlSN5	
SlSN9	SlSN3		
SlSN10	SlSN4		
	SlSN6		
	SlSN7		
	SlSN8		
	SlSN9		
	SlSN10		
	SlThi3		
SlLTP1.4	SlLTP1.1	SlLTP1.5	
SlLTPd3.1	SlLTP1.2	SlLTP2.1	
SlLTPd6.5	SlLTP1.5	SlLTPd6.1	
SlLTPg1.1	SlLTPd2.2	SlLTPd6.2	
SlLTPg2.4	SlLTPd2.3	SlLTPd6.3	
SlLTPg2.6	SlLTPd6.1	SlLTPd6.6	
SlLTPg2.7	SlLTPd6.3	SlLTPd6.9	
SlLTPx2.1	SlLTPd6.5	SlLTPd6.10	
	SlLTPd6.6		
	SlLTPd6.8		
	SlLTPd6.9		
	SlLTPd6.10		
	SlLTPg2.6		
	SlLTPg2.9		
SlHev1			SlHev1
	SlKnot1		
	SlKnot2		
	SlRALF2		
	SlRALF5		
	SlRALF6		
	SlRALF7		
SlOlee1.4			
SlOlee6.2	SlOlee6.2		
			SlEPF3
	SlCRP1		
SlPR-1.2	SlPR-1.1	SlPR-1.5	SlPR-1.6
SlPR-1.3	SlPR-1.2	SlPR-1.6	SlPR-1.7
	SlPR-1.4		SlPR-1.8
			SlPR-4.1

[1] Up-regulated genes are those with an expression fold change ≥2, down-regulated genes are those with an expression fold change ≤0.5. [2] Differentially expressed genes in IR-expressing plants compared to those treated with the elicitors. [3] Differentially expressed genes in IR-expressing tomato plants compared to those infected with *F. oxysporum*. Genes up-regulated in both variants are highlighted yellow. Genes down-regulated in both variants are highlighted green.
