# Supplementary material for: Molecular Insights into the Role of Cysteine-Rich Peptides in Induced Resistance to Fusarium oxysporum Infection in Tomato Based on Transcriptome Profiling
Source: Int J Mol Sci. 2021 May 27;22(11):5741. doi: 10.3390/ijms22115741 (PMC8198727; doi:10.3390/ijms22115741)
Supplement: Supplementary file 1 [file ijms-22-05741-s001.zip › Table S7.pdf]

Table S7. List of primers for RT-PCR validation.

№	Gene	Pr_dir 5' –> 3'	Pr_rev 5' –> 3'	Length (bp)	T primer annealing (°C)
1.	*SlSN1*	TCTCTAACGTGGTCGAGGGT	AGGGCATGATTGTTTGTTGCC	176	59
2.	*SlSN7*	TGTTCAAAGGCAGGACGACA	AGGACATTTAGGCCCTCCCT	133	59
3.	*SlRALF1*	AGGGGAGTATTGCAGAGTGC	TTGAGCTCCAGGTTTGCAGT	173	59
4.	*SlRALF7*	AGGTGCAACAAGTAACTGAAGC	CCGTCGCGTCGAAAGATAGA	120	59
5.	*SlMEG2*	CATGCTGCCCCAATTTCACAA	GCAAGCATACTGCTTTAGCCC	169	60
6.	*SlOlee1.2*	TGCCAAGATCCTTGGAGGTC	ACTCTTGTTGGGTGAAGGGC	148	60
7.	*SlThi2*	TTCGTGTGCGAGAGAGTCTG	TGACACAGACAGTACGAGCG	151	61
8.	*SlDEFL2*	TGCTTGTCATGGCTACTGGAC	GGTCTCACATACCGAGGCAC	119	62
9.	*SlDEFL8*	CCTACTGGTGTGGTTGTCCC	GGACCCTCCATTTCCCACAG	86	59
10.	*SlKnot1*	GCTGCTCAAGATGTGATGGC	TCCAACAGGCCTGACAGAAC	130	59
11.	*SlLTPd6.8*	AATGGCCAAGTTTGGTGCAAC	CAAGGTGCACTAAGTCATTTAGC	251	60
12.	*SlLTP1.5*	AAGCCTTAAGCTGTGGGCAA	GAAGATCAGCAGCTTTGCGG	210	61
13.	*SlLTP2.3*	TTGCCTTAGCGCGATCACAT	GGGGTAGGGAACACCACAAG	169	59
14.	*SlCRP1*	TGTTCCTTGCTATGTTTGTGTTGA	GGCAAGGCACAAAGTTAGGC	128	60
15.	*SlEPF1*	GGGTTCGATGCCACCAAGTT	TCTAGTGGTGTGGTGCATGG	151	62
16.	*EF1-α*	ACCTTTGCTGAATACCCTCCATTG	CACAGTTCACTTCCCCTTCTTCTG	150	59-62
17.	*SlHev1*	TGGGCATGATCACACCCTAT	AAGCCTACAACACAACGTCT	431	58
